# Supplementary material for: Evolution of Collective Behaviors for a Real Swarm of Aquatic Surface Robots
Source: PLoS One. 2016 Mar 21;11(3):e0151834. doi: 10.1371/journal.pone.0151834 (PMC4801206; doi:10.1371/journal.pone.0151834)
Supplement: S1 Text — Document detailing the aquatic robotic platform and the experimental parameters of both the simulated and real experiments, in some cases repeating and extending the information given in the main manuscript. (PDF) [file pone.0151834.s001.pdf]

Evolution of Collective Behaviors for a Real Swarm of Aquatic Surface Robots — Supporting Information

Miguel Duarte[1,2,3,*], Vasco Costa[1,2,3], Jorge Gomes[1,2,4], Tiago Rodrigues[1,2,3], Fernando Silva[1,2,4], Sancho Moura Oliveira[1,2,3], Anders Lyhne Christensen[1,2,3]

1 BioMachines Lab, Lisbon, Portugal
2 Instituto de Telecomunicações, Lisbon, Portugal
3 University Institute of Lisbon (ISCTE-IUL), Lisbon, Portugal
4 BioISI, Faculdade de Ciências, Lisbon, Portugal

*** miguel_duarte@iscte.pt**

Experimental Details

Robotic Platform

We developed and produced a total of 10 relatively small (60 cm) and inexpensive ($\approx$ 300 EUR/unit) robots. We used digital manufacturing techniques to produce the robots, such as fused deposition 3D printing and CNC milling. Furthermore, we used widely available and off-the-shelf hardware in order to keep costs low, see Table 1 and Fig. 1. The robot is a differential drive monohull boat and its physical and dynamic properties are presented in Table 2. The Raspberry Pi 2 single-board computer was used for the control unit of each robot, and communication is achieved using an ad-hoc wireless network. A Kalman filter was applied to the GPS and compass readings of the real robots before they are used to compute sensory readings for the controller. Schematics, 3D models, and source code are available at `http://biomachineslab.com/aquaticdrone`.

Robots communicate with neighboring robots and with a base station using a IEEE 802.11g based ad-hoc wireless network (Wi-Fi). In order to assess the range of the chosen wireless adapter, we conducted empirical tests with the robots floating on the water surface, and achieved communication up to 40 m. When the swarm of robots is deployed, inter-robot communication is achieved by broadcasting messages. Each robot transmits a short status message, indicating its identification, position, and orientation. The status message is broadcast every second allowing neighboring robots to sense one another.

In order to monitor the swarm, an application monitored the messages that were sent by the robots in the swarm and displayed the locations in a map. To increase the range at which the inter-robot communication could be eavesdropped, an Ubiquiti BULLET-M2-HP was used at the base station, effectively increasing the monitoring range up to 300 m. The application was also used to send commands and messages to the robots. Examples include starting or stopping a specific controller, sending a list of waypoints or a geo-fence, and updating the onboard control software.

Experimental Parameters

Table 3 lists the parameters used in our experiments, both in the simulation environment and in the real experiments. The noise that was applied in simulation during evolution is described below. All random numbers were drawn from uniform distributions. Regarding

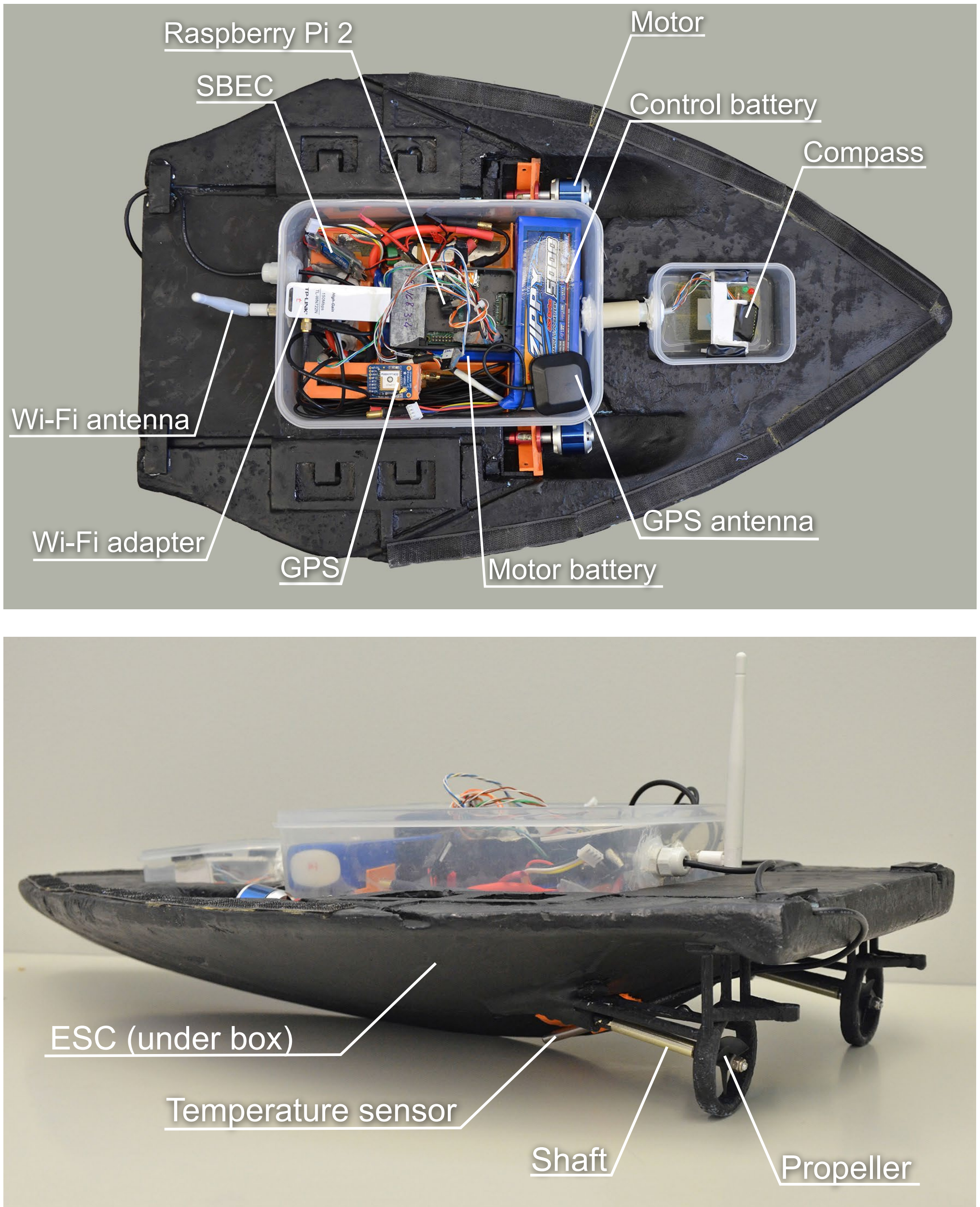

Figure 1. Top and side view of the final robot prototype with a description of the components.

the movement model of the simulated robot, the values were taken by systematically performing tests with the real robot at different speeds and headings. The simulated dynamics were implemented taking into account the measurements obtained from these tests, and match the physical properties described in Table 2.

GPS noise: upper limit for noise added to the robots' GPS unit at every simulation timestep. The value was taken from the technical specification of the GPS used in our robots.

Compass noise: upper limit for noise added to the robot's compass at every simulation timestep, chosen from empirical tests with the LSM303D compass.

Table 1. Robotic platform component list.

Component	Make & Model
Motors (A)	NTM Prop Drive Series 28-30 A 750 kv / 140w
Motors (B)	Emax 2215/25 950 kv 2-3S
Shaft	4 mm drive shaft
Shaft sleeve	255 mm boat shaft sleeve
Propellers	3-blade 28 mm
ESC	HobbyKing 50 A Boat ESC
Control battery	Zippy 40C Series 5000 mA 3 S LiPo
Motor battery	Zippy 30C Series 8000 mA 3 S LiPo
GPS	Adafruit Ultimate GPS Breakout
Compass	STMicroelectronics LSM303D
Water temperature sensor	DS18B20
Onboard computer	Raspberry Pi 2
Wi-Fi adapter	TP-Link TL-WN722N
Hull material	Extruded Polystyrene (XPS) + fiberglass with epoxy resin
Structural components	3D printed Polylactic Acid (PLA)
Electronics enclosure	2.5 L watertight plastic box
Compass enclosure	0.4 L watertight plastic box

Table 2. Measured movement dynamics and physical properties.

Parameter	Value	Parameter	Value
Size (L × W × H)	65 × 40 × 15 cm	Weight	3 Kg
Minimum speed	0.3 m/s	Maximum speed	1.7 m/s
Maximum turning radius	90 °/s	Maximum acceleration	1.7 m/s^2
Time from full speed to stop	5 s	Autonomy (at full speed)	1h30m

Motor delay: fixed delay between executing a motor speed command and observing a reaction in the movement of the robot.

Heading offset: upper limit for noise added to the heading of the robot. The value is set individually for each robot at the beginning of a sample.

Speed offset: upper limit for noise added to the speed of the robot. The value is set individually for each robot at the beginning of a sample.

Motor output noise: upper limit for noise added to the output of the controllers at every simulation step.

Drift speed: upper limit for the translation component added to all robots. The drift is intended to simulate the effects of water currents and wind. The value is chosen at the beginning of each sample, along with a random orientation, and is added to the position of all robots at every simulation timestep.

Table 3. Parameters used in the experiments.

Parameter	Value	Parameter	Value
**NEAT**			
Population size	150	Target species count	5
Recurrency allowed	true	Mutation prob.	25%
Prob. add node	3%	Prob. mutate bias	30%
Prob. add link	5%	Crossover prob.	20%
**Simulation noise**			
GPS noise	1.8 m	Compass noise	10°
Motor delay	500 ms	Heading offset	5%
Speed offset	10%	Motor output noise	5%
Drift speed	[0,0.1] m/s		
**Homing task**			
Generations	100	Trial length (evolution)	100 s
Deploy area (evo.)	50 m × 50 m	Waypoint distance[1] (evo.)	[0,50] m
Robot sensor range	20 m	Waypoint sensor range	10 m
Initial robot separation	> 5 m	Trial length (real)	240 s
Number of waypoints[2] (real)	4	Distance between WPs (real)	40 m
**Dispersion task**			
Generations	100	Trial length (evo.)	100 s
Deploy area (evo.)	30 m × 30 m	Target distance[3]	20 m
Robot sensor range	40 m	Initial robot separation	> 5 m
Trial length (real)	90 s	Deploy area, 4 robots (real)	20 m × 20 m
Deploy area, 6 robots (real)	24 m × 24 m	Deploy area, 8 robots (real)	28 m × 28 m
**Clustering task**			
Generations	400	Trial length (evo.)	200 s
Initial robot separation	[20,40] m	Robot sensor range	40 m
Deploy area	100 m × 100 m	Clustering threshold[4]	7 m
Trial length (real)	180 s		
**Area monitoring task**			
Generations	100	Trial length (evo.)	200 s
Robot sensor range	40 m	Geo-fence sensor range	40 m
Monitoring area (evo.)	[0.5,1.7] ha	Deploy area	1.44 ha
Initial robot separation	> 5 m	Trial length (real)	300 s
Monitoring area (real)	1 ha	Deploy area (real)	1 ha

[1] Distance from the center of the deploy area.
[2] In the real experiments, the robots had to navigate through a sequence of waypoints.
[3] Distance that the robots should maintain from each other.
[4] Maximum distance for two robots to be considered as part of the same cluster.
